# Progranulin improves neural development via the PI3K/Akt/GSK-3β pathway in the cerebellum of a VPA-induced rat model of ASD

**DOI:** 10.1038/s41398-022-01875-4

**Published:** 2022-03-22

**Authors:** Lili Wang, Jianhui Chen, Yuling Hu, Ailing Liao, Wenxia Zheng, Xiaoqing Wang, Junying Lan, Jingjing Shen, Shali Wang, Feng Yang, Yan Wang, Yingbo Li, Di Chen

**Affiliations:** 1 Cerebrovascular Diseases Laboratory, Institute of Neuroscience, Chongqing Medical University, Chongqing, 400016 China; 2 Advanced Innovation Center for Human Brain Protection, Capital Medical University, Beijing, 100070 China; 3 China National Clinical Research Center for Neurological Diseases, Beijing Tiantan Hospital, Capital Medical University, Beijing, 100070 China; 4 Present Address: Qujiang No. 2 Middle School, Xi’an, 710000 China; 5 Present Address: Department of Nuclear Medicine, Nanchong Central Hospital, The Second Clinical College of North Sichuan Medical College, Nanchong, 637000 China

**Keywords:** Molecular neuroscience, Pharmacodynamics

## Abstract

Autism spectrum disorder (ASD) is a neurodevelopmental disease featuring social interaction deficits and repetitive/stereotyped behaviours; the prevalence of this disorder has continuously increased. Progranulin (PGRN) is a neurotrophic factor that promotes neuronal survival and differentiation. However, there have not been sufficient studies investigating its effect in animal models of autism. This study investigated the effects of PGRN on autistic phenotypes in rats treated with valproic acid (VPA) and assessed the underlying molecular mechanisms. PGRN was significantly downregulated in the cerebellum at postnatal day 14 (PND14) and PND35 in VPA-exposed rats, which simultaneously showed defective social preference, increased repetitive behaviours, and uncoordinated movements. When human recombinant PGRN (r-PGRN) was injected into the cerebellum of newborn ASD model rats (PND10 and PND17), some of the behavioural defects were alleviated. r-PGRN supplementation also reduced cerebellar neuronal apoptosis and rescued synapse formation in ASD rats. Mechanistically, we confirmed that PGRN protects neurodevelopment via the PI3K/Akt/GSK-3β pathway in the cerebellum of a rat ASD model. Moreover, we found that prosaposin (PSAP) promoted the internalisation and neurotrophic activity of PGRN. These results experimentally demonstrate the therapeutic effects of PGRN on a rat model of ASD for the first time and provide a novel therapeutic strategy for autism.

## Introduction

Autism spectrum disorder (ASD) comprises a group of neurodevelopmental conditions characterised by impairment in social interaction, verbal communication, and repetitive, stereotyped behaviours [1]. The prevalence of ASD diagnosis has increased markedly in recent years, and thus, the disorder has emerged as a tremendous public health concern. Despite the high incidence of ASD, the precise aetiologies of ASD remain unknown. Complex causes with genetic, immunological, inflammatory, metabolic, and environmental factors are related to ASD. There is currently no effective treatment, pharmacological or otherwise, that alleviates the core behaviours that characterise ASD; existing treatments merely target comorbid conditions such as anxiety, depression, seizures, and attention-deficit/hyperactivity disorder (ADHD) [2, 3]. Gaining mechanistic insight would thus provide better entry points for the development of curative interventions and therapeutics for this disorder.

The cerebellum is well-known for its role in mediating motor coordination and complex motor behaviour. Nevertheless, recent evidence suggests that the cerebellum may also play an essential and decisive role in emotional behaviours and cognitive functions [4, 5]. It is implicated in higher-order cognitive and affective processes through different cortico-cerebellar loops [6]. Meanwhile, growing evidence shows that cerebellar dysfunction is closely related to various psychiatric disorders, including ASD [7, 8]. For example, postmortem studies have demonstrated that cerebellar pathology is among the most consistent findings in ASD patients [9]. Isolated injury of the early postnatal cerebellum could contribute to an increased incidence of ASD [10]. Furthermore, previous studies have revealed early cerebellar hyperplasia [11] and hypoplasia of vermis lobules VI–VII [12] in ASD. Collectively, these findings demonstrate that changes in the cerebellum play an essential role in the pathophysiology of ASD.

Progranulin (PGRN), also known as granulin/epithelin precursor, acrogranin, or prostate cancer cell-derived growth factor, is a secreted glycosylated protein with seven and a half cysteine-rich repeats [13]. As a broadly expressed pleiotropic protein, PGRN participates in several biological pathways, including embryogenesis, tumorigenesis, inflammation, and host defence. It has also been identified as a neurotrophic factor promoting neurite outgrowth, neuronal survival, and differentiation [14]. Importantly, in addition to its association with the pathogenesis of frontotemporal lobar degeneration (FTLD), which is universally recognised in the field [15], PGRN plays a crucial role in the occurrence of ASD. Low plasma PGRN levels have been found in a group of patients with autism [16]. Furthermore, loss of synaptic zinc transport in PGRN-deficient mice may contribute to autism-like behaviours and chronic pain [17]. On the other hand, in the central nervous system, the expression of PGRN is restricted to specific neuronal populations during the late stage of development, and the Purkinje cell layer in the cerebellum is one of the regions with strong expression of PGRN [18, 19]. It has been demonstrated that global PGRN knockout mice exhibit changes in Purkinje cell dendrites and abnormal locomotor activity [20]. There is also evidence that PGRN retrogradely strengthens climbing fibre (CF) synaptic inputs and counteracts redundant CF synapses in the developing mouse cerebellum [21]. Therefore, the abnormal expression of PGRN may disturb synapse outgrowth in the cerebellum and increase the risk of developing neuropsychiatric disorders such as ASD. However, the downstream signalling mechanisms of PGRN are not fully understood.

In the current study, immunofluorescence analysis and Western blotting were used to investigate the expression of PGRN in the cerebellum of VPA-exposed offspring. Then, we evaluated whether human recombinant PGRN (r-PGRN) reversed autism-like behaviours and motor incoordination and affected neuronal apoptosis and synaptic formation/elimination. Furthermore, we examined whether PGRN modulated phosphoinositide-3-kinase/Akt/glycogen synthase kinase 3 beta (PI3K/Akt/GSK-3β) signalling by injecting the PI3K inhibitor wortmannin to block the downstream signalling pathway in the cerebellum. Meanwhile, we found that PGRN might be regulated by the prosaposin (PSAP) pathway in the rat autism model. These findings thus provide new insight into the roles of PGRN in ASD and support a mechanistic basis for the targeted therapy of autism-like behaviours.

## Materials and methods

### Reagents and antibodies

Valproic acid (VPA), pentobarbital solution, Dimethyl sulfoxide (DMSO) and 4′,6-diamidino-2-phenylindole (DAPI) were purchased through Sigma, USA; Restructuring PGRN (r-PGRN) was from Sino Biological, China; phosphoinositide-3-kinase (PI3K) inhibitor wortmannin was from Cell Signalling Technology, USA; BCA Assay Kit, One Step TUNEL Apoptosis Assay Kit, horseradish peroxidase (HRP)-labelled secondary antibody, 5% bovine serum albumin (BSA) and fluorescent secondary antibody were procured from Beyotime, China; Immobilon® Western Chemiluminescent HRP Substrate was from Millipore, USA; Progranulin ELISA kit was procured from Jiangsu Jingmei Biological Technology Co., Ltd., China; The Golgi-Cox Standard Kit was purchased through HITO, USA. The antibodies used were anti-PGRN (catalogue: 18410-1-AP; Proteintech, China), anti-Caspase-3 (catalogue: 19677-1-AP; Proteintech, China), anti-Bcl-2 (catalogue: ab196495; Abcam, UK), anti-Bax (catalogue: 50599-2-Ig; Proteintech, China), anti-postsynaptic density protein 95 (PSD95) (catalogue: 20665-1AP; Proteintech, China), anti-synaptophysin (SYP) (catalogue: 17785-1-AP; Proteintech, China), anti-phosphorylated-Akt (ser473) (catalogue: AF0016; Affinity Biosciences, USA), anti-Akt (catalogue: 10176-2-AP; Proteintech, China), anti-phosphorylated-GSK-3β (ser 9) (catalogue: bs-2066R; Bioss Biotechnology, China), anti-GSK-3β (catalogue: bsm-33293M; Bioss Biotechnology, China), anti-mannose 6-phosphate receptor (M6PR) (catalogue: ab124767; Abcam, UK), anti-sortilin (SORT1) (catalogue: ab16640; Abcam, UK), anti-prosaposin (PSAP) (catalogue: A1819; ABclonal, China), anti-GADPH (catalogue: AF7021; Affinity Biosciences, USA) and anti-β-actin (catalogue: 20536-1-AP; Proteintech, China).

### Animals

Eight-week-old Sprague–Dawley rats (Chongqing Medical University, China) were used in our study. For the VPA-induced animal model of autism (VPA-exposed rat or VPA rat), prenatal exposure to VPA or saline was performed as previously reported [22]. The pregnant rats were treated with a single intraperitoneal injection of either sodium valproate (250 mg/ml dissolved in sterile normal saline) or sterile normal saline at a dose of 600 mg/kg. Offspring (males) were retained and randomly divided into four experimental groups, including the control group (CON), VPA-treated group (VPA + Vehicle), VPA + r-PGRN group (r-PGRN), and VPA + r-PGRN + wortmannin group (r-PGRN + Wort). On postnatal days (PND) 10 and PND17, we used brain stereotaxic guidance to administer different reagents into the cerebellum. Stereotaxic surgeries were completed under pentobarbital anaesthesia (40 mg/kg, ip), and rats were mounted onto a stereotaxic apparatus (Catalogue No. 78-8130, RIWARD, China). A microlitre syringe (5 μl) was inserted into each side of the cerebellum successively (2.5 mm posterior to lambda, 2.5 mm lateral to the midline, and 3.0 mm ventral to the surface of the skull). In the control group, this syringe was used to inject 2.5 μl sterile normal saline on each side; the needle was slowly withdrawn from each target site 10 min after injection. The VPA + Vehicle group received 5% dimethyl sulfoxide (DMSO) diluted with sterile normal saline at the same volume. We injected r-PGRN (2.5 μl per side, 50 ng/μl dissolved in 5% DMSO) into the cerebellum. The PI3K inhibitor wortmannin (1 μl each side, dissolved at 2 mM in DMSO, and diluted 20 times with sterile normal saline before use) was administered before the injection of r-PGRN for 30 min. Offspring rats were weaned at PND21 and then selected for behavioural tests beginning at PND28. The subjects were subjected to behavioural tests until PND35, after which the rats were euthanized, and the brains were collected for biochemical assays. Rats were allowed ad libitum to feed, water, breed, and nurture their offspring under normal circumstances. All experimental animal programs were carried out under the guidelines of the *Chongqing Management Approach of Laboratory Animals* (Chongqing government order No. 195). Ethical approval was obtained from the Ethics Committee of Chongqing Medical University.

### Behavioural tests

All the behavioural tests were recorded with a camera (Aoni, China), and the results were analysed by a researcher blinded to the experimental group.

#### Open field test and self-grooming test

A transparent acrylic box (100 × 100 × 40 cm) was used for this exploratory and locomotor activity test. The box floor was divided into 25 equal squares, with 9 squares composing the centre grid. Each rat was given 10 min to adapt to the arena before the start of recording. Then, they were placed in the central zone of the box and allowed to explore freely for 10 min. The number of squares that rats passed through horizontally (surrounding and centre grid squares crossed) and the frequencies of rearing and climbing walls (vertical scores) were recorded. Simultaneously, the duration time of stereotyped movements (self-grooming) and the defecation frequency of rats were also counted. After each trial, the apparatus was thoroughly cleaned with 75% alcohol and allowed to dry.

#### Juvenile social play

The transparent acrylic box (100 × 100 × 40 cm) described above was used to line the box with clean bedding. Before the test, the subject rat was allowed to roam freely in the box for 10 min. Subsequently, a stranger rat from another box of the same age and sex was introduced into the box. During the test, the interaction time (sniffing, touching, licking, and following the stranger) and the number of times the subject attacked the stranger and dug the bedding were measured.

#### Three-chamber social test

The apparatus was a rectangular transparent acrylic box (120 × 45 × 40 cm) separated into three chambers of equal size. Identical cages were placed in each side chamber. In general, the testing process consisted of three sessions. First, the subject rat was individually accommodated for 10 min in the box before the experiment. In the second 10 min session of the sociability phase, a stranger rat was introduced into the left wire cage (Stranger 1), and the other wire cage was empty (Object). The chamber with stranger 1 was named chamber A, and the other chamber was named chamber B. In the last 10 min session of the social preference phase, another stranger rat was placed in the right wire cage (Stranger 2). The subject rat was free to explore the three chambers during all test times. The time spent in each chamber and olfactory communication with the strange rat or object were recorded to assess sociability and social preference. Before each trial, the apparatus was thoroughly cleaned with 75% ethanol to minimise residual rat odours.

#### Righting reflex

Briefly, rats were held 10 cm above the desktop in the supine position, and the experimenter withdrew her hands as simultaneously and quickly as possible. Their latency time to turn over to the upright position and all paws in contact with the platform was measured. Each subject was given three trials/day in succession. The trials were digitally recorded by a camera (Aoni, China) at a rate of 35 frames/s. Then, we reduced the original speed to assess reflexes through Adobe Premiere frame by frame.

#### Static beams

In this test, a specific beam consists of a 60 cm long wooden rod (1.5 cm diameter) placed horizontally 40 cm above the floor. Animals were habituated to the apparatus and trained to traverse the wooden beam one day before the test. After the training day, rats were given three trials to cross the beam. The time to cross the beam without falling off and the number of hind paw slips were measured. If a rat could not complete the beam run (stationary) or fell from the beam, the test was finished at 40 s (cut-off time). Meanwhile, we calculated the success rate of the rats traversing the wooden beam (proportion of trials).

#### Gait analysis

Each rat’s fore and hind paws were painted nontoxic red and blue ink, respectively, before allowing them to traverse a sheet of white paper (10 cm × 60 cm). Each animal was given three recorded trials for footprint analysis. We measured front paw stride length and width and hind paw stride length from each set of paw prints left. Measurements in a single set were averaged, and the means were used in the data analysis.

#### Traction test

The rats were suspended by forepaws on a steel wire (2 mm in diameter and 120 cm in length) stretched horizontally 50 cm over the floor. The length of time the rats could hang on the wire until it fell was recorded. Meanwhile, we scored their hanging posture to assess the balance of the rats. The hind paw placements of the rat were scored into 1–3 points: 1 or 2, Neither or one of two hind paws gripped the wire; and 3, both hind paws gripped the wire. The test was conducted three times with an inter-trial interval of approximately 20 min. Mean measurement over the three tests was analysed.

### Western blot analysis

Protein concentrations were estimated with a BCA assay. Equal concentrations of protein (20 μg) were loaded on 8–10% sodium dodecyl sulfate-polyacrylamide gel electrophoresis gels and then transferred to PVDF membranes. Subsequently, the membranes were blocked with 5% skim milk for 2 h and then incubated overnight at 4 °C with the following primary antibodies: anti-PGRN (1:1000), anti-Bax (1:8000), anti-Bcl-2 (1:500), anti-Caspase-3 (1:1500), anti-PSD95 (1:2000), anti-SYP (1:2000), anti-p-AKT (Ser473) (1:2000), anti-AKT (1:2000), anti-p-GSK-3β (Ser9) (1:2000), anti-GSK-3β (1:2000), anti-M6PR (1:100000), anti-SORT1 (1:1000), anti-PSAP (1:2000), and anti-β-actin (1:2000). Then, the membranes were washed with TBST and incubated with HRP-labelled secondary antibody for 1 h. Immobilon^®^ Western Chemiluminescent HRP Substrate was used to detect the protein bands. The immunoreactive bands were scanned and densitometrical analysed by ImageJ software.

### TUNEL assay

Rats were perfused intracardially with saline followed by 4% paraformaldehyde under deep sodium pentobarbital anaesthesia. The brains were rapidly harvested and postfixed in 4% paraformaldehyde solution for 24 h and dehydrated in increasing concentrations (10%, 20%, and 30%) of sucrose solution for 3 days at 4 °C. Then, brains were sectioned into 10-μm-thick coronal slices with a frozen microtome (Leica, Germany) for the following experiment. Brain tissues were stained to detect apoptosis of neurons according to the manufacturer’s recommended instructions of the One Step TUNEL Apoptosis Assay Kit. Nuclei were counterstained with 4′,6-diamidino-2-phenylindole (DAPI), and the cells were observed with a fluorescence microscope (Leica, Germany). The apoptotic index (AI) was defined as follows: AI = number of positive cells/a total number of cells × 100%.

### Immunofluorescence

The preparation of brain sections is described above. The sections were incubated in 0.5% Triton-X 100 for 30 min and then blocked in 5% BSA for 1 h at room temperature. Subsequently, the sections were incubated in the following primary antibodies overnight at 4 °C: anti-PGRN (1:200), anti-PSD95 (1:200) and anti-SYP (1:200). On the second day, the sections were rewashed with phosphate-buffered saline (PBS) and incubated with a fluorescent secondary antibody for 1 h. Finally, the sections were counterstained with DAPI. A fluorescence microscope (Leica, Germany) and ImageJ software were used to measure the average density (IOD/area).

### Enzyme-linked immunosorbent assay (ELISA)

To quantify cerebellar PGRN expression levels, we used a commercial Progranulin ELISA kit (Jingmei, China) according to the instructions provided by the manufacturer. Chopped brain tissue was homogenised in PBS buffer (1 g tissue + 9 ml PBS) and centrifuged for 20 min (4 °C) at 3000 rpm. The supernatants were used for analysis. First, 50 μl of standards was dispensed into corresponding wells, and equal brain lysate samples diluted into the diluent buffer at 1:5 were also dispensed into appropriate wells. Then, they were mixed with enzyme conjugate reagent and incubated at 37 °C for an hour. After washing, 50 μl of colour A and colour B reagent were added. The reaction was allowed to proceed for 15 min in the dark to develop a blue colour, whose intensity reflected the quantity of PGRN. The reaction was finished by the addition of a 50 μl stop solution. All samples were assayed in triplicate and measured at 450 nm to read the optical density. The results were converted into pg/ml based on the standard curve.

### Golgi-Cox analysis

Brains used for Golgi-Cox staining were sectioned on a vibratome in the sagittal plane at 100 μm. Brains were processed for Golgi stained using the Golgi-Cox OptimStain^TM^ PreKit as directed by the manufacturer after dried in the dark at room temperature. The Purkinje cells in the cerebellar were imaged using a bright-field microscope (Leica, Germany). Ten Purkinje neurons from each cerebellum per group were analysed of spine density using the ImageJ analysis system. For each Purkinje neuron, we randomly selected five different dendritic secondary and tertiary branches for the number of spines.

### Statistical analysis

The results are expressed as the mean ± SEM. Statistical analysis of the data was performed with GraphPad Prism software (GraphPad Prism 8). The sample size was chosen according to similar experiments and G power analysis. First, all data were assessed for normality and homogeneity of variance, and the statistical test was chosen accordingly. The independent-sample *t* test or Mann–Whitney test was used to analyse the data between two groups. If the condition of equal variances was not met, Welch’s correction was used. Multi-group comparisons were analysed with one-way analysis of variance (ANOVA) followed by post hoc Tukey’s multiple comparisons. For data that were not normally distributed, the Kruskal–Wallis test was used for post hoc pairwise comparison. Two-way ANOVA with Sidak post hoc comparison was used to compare the effects of prenatal treatment and time. A value of *P* < 0.05 was considered statistically significant.

## Results

### Prenatal exposure to VPA caused alterations in PGRN temporal expression in the cerebellum and behavioural abnormalities

**Fig. 1:**
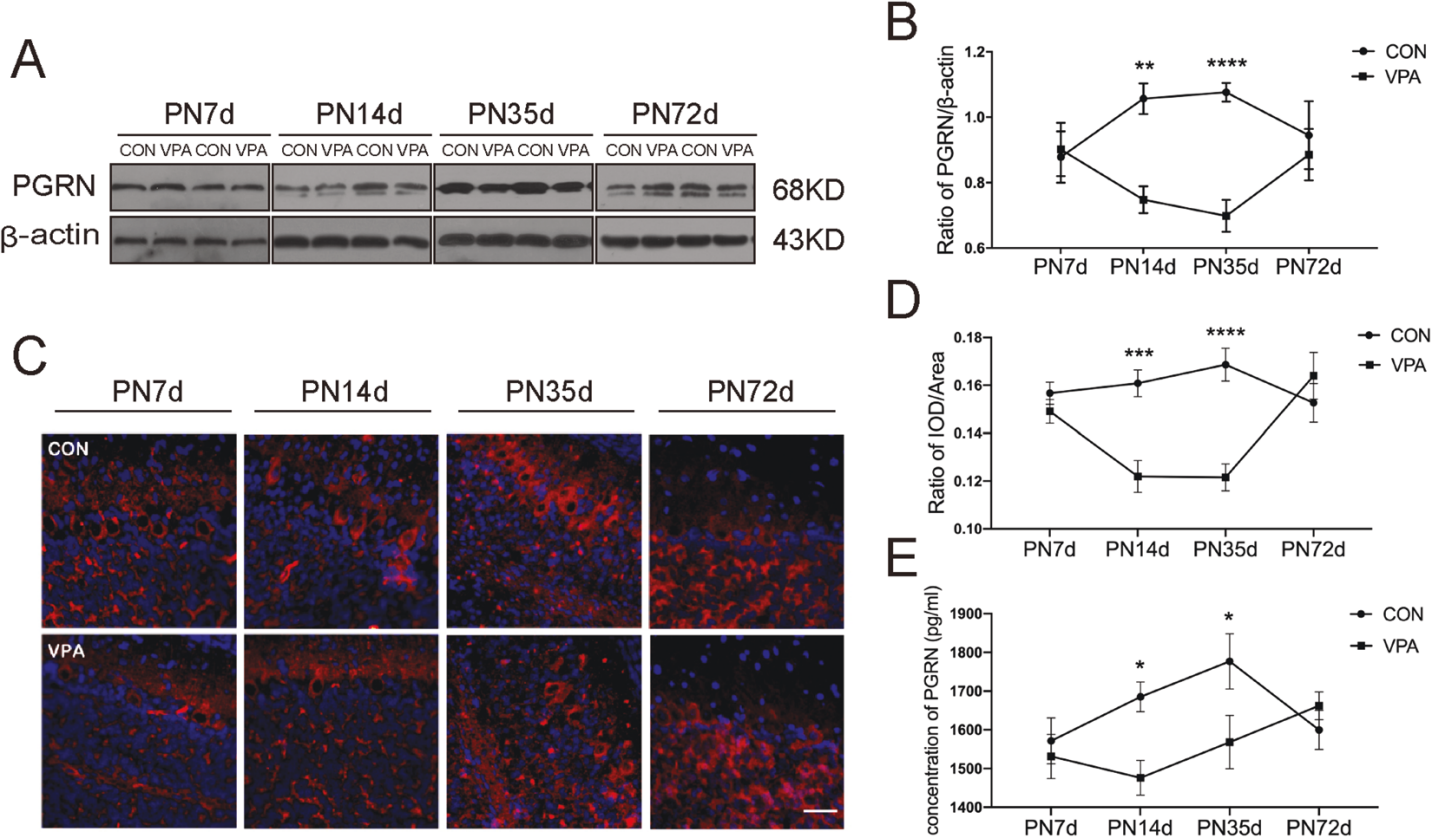
Prenatal exposure to VPA caused the alterations of PGRN temporal expression in the cerebellum. Representative blots (**A**) and quantification (**B**) showed the expression of PGRN at four different time points. Representative images (bar = 100 μm) (**C**) and quantification (**D**) of immunofluorescence staining described that the expression of PGRN is in the same trend as Western blotting. **E** PGRN ELISA protein quantitation of cerebellar tissue. Data are expressed as mean ± SEM. Two-way ANOVA followed by Sidak post hoc. (*P < 0.05, **P < 0.01, ***P < 0.001, ****P < 0.0001, sample sizes (*n*): *n* = 5/group for Western blotting and immunofluorescence staining. *n* = 4/group for ELISA).

To explore whether prenatal VPA exposure-induced changes in PGRN expression in the cerebellum, we used Western blotting, immunofluorescence staining and ELISA to examine PGRN protein expression in the control and VPA groups at four different time points. Western blotting analysis showed that PGRN expression was significantly decreased at PND14 and PND35 (*F*_(prenatal treat)1,126_ = 15.57, *P* = 0.0001, post hoc, *P* = 0.0035 for PND14, *P* < 0.0001 for PND35, Fig. 1A, B). Meanwhile, the results detected in immunofluorescence were in line with the Western blotting analysis (*F*_(prenatal treat)1,104_ = 18.65, *P* < 0.0001, post hoc, *P* = 0.0003 for PND14, *P* < 0.0001 for PND35, Fig. 1C, D). More importantly, we used a more quantitative method, such as ELISA, to verify our experimental results (*F*_(prenatal treat)1,108_ = 6.268, *P* = 0.0138, post hoc, *P* = 0.0137 for PND14, *P* = 0.0263 for PND35, Fig. 1E). These results suggest that prenatal exposure to VPA could lead to the low expression of PGRN in the cerebellum at specific stages of development.

**Fig. 2:**
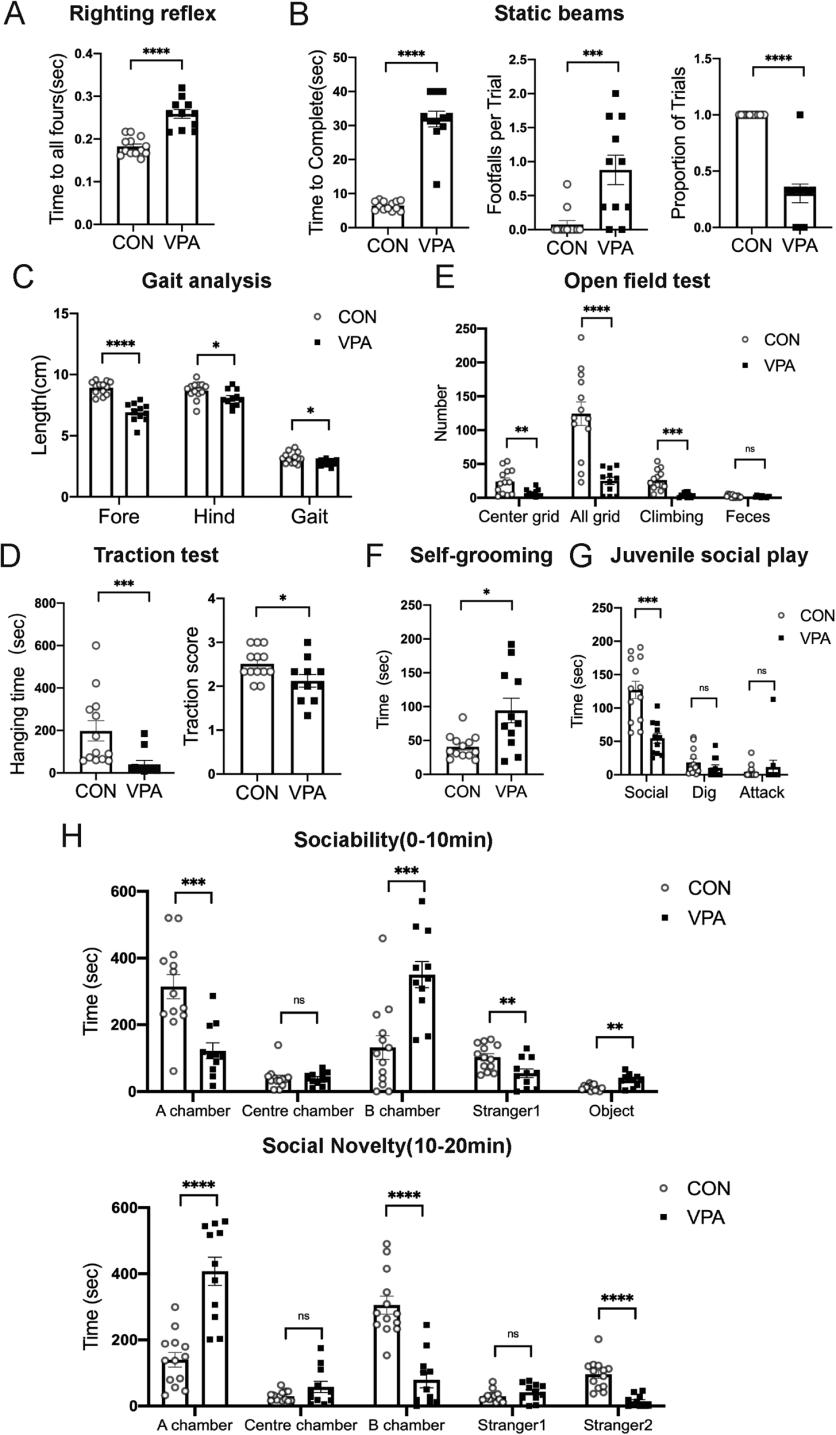
Prenatal exposure to VPA induced the defection of behaviours. Cerebellum-related behaviours: righting reflex (**A**), static beams (**B**), gait analysis (**C**), traction test (**D**). Autism-like behaviours: open field test (**E**), self-grooming test (**F**), juvenile social play (**G**), the three-chamber social test (**H**). Data are expressed as mean ± SEM. Student’s *t*-test (A, C_(Fore, Hind, Gait)_, D_(Traction score)_, E_(Faeces)_, G_(Social)_, H_(10 min: Stranger1, A chamber, B chamber)_, H_(20 min: A chamber, B chamber)_); Mann–Whitney test (B_(Time to Complete, Footfalls per Trial)_, D(_Hanging time)_, G_(Dig, Attack)_, H_(10 min: Centre chamber)_, H_(20 min: Stranger1, Stranger2)_); Welch’s correction (B_(Proportion of Trials)_, E_(Centre grid, All grid, Climbing)_, F, H_(10 min: Object)_, H_(20 min: Centre chamber)_). (ns no significant difference, *P < 0.05, **P < 0.01, ***P < 0.001, ****P < 0.0001, Sample sizes (*n*): *n* (CON) = 13, *n* (VPA) = 11).

We next examined the motor performance of VPA-exposed rats with a series of tasks related to the cerebellum. The righting reflex results showed that VPA-treated rats spent more time adjusting from the supine position to standing on all four limbs than control rats (*t* = 6.626, d*f* = 22, *P* < 0.0001, Fig. 2A). In the static beam test, the VPA group performed significantly worse: it exhibited prolonged traversing time (Mann–Whitney *U* = 0, *P* < 0.0001), increased times of hind paw slips (Mann–Whitney *U* = 19.50, *P* = 0.0009), and a lower success rate (Welch-corrected, *t* = 8.352, d*f* = 10.00, *P* < 0.0001, Fig. 2B). Gait analysis showed that the VPA group had significantly shorter gaits than the control group (Fore: *t* = 7.550, d*f* = 22, *P* < 0.0001; Hind: *t* *=* 2.219, d*f* = 22, *P* = 0.0371; Gait: *t* = 2.551, d*f* = 22, *P* = 0.0182, Fig. 2C). In the traction test, the hanging time of the VPA group was meaningfully shorter than that of control rats (Mann–Whitney *U* = 15, *P* = 0.0005). Moreover, the traction scores of VPA-treated rats were obviously below the control group according to the traction score standard (*t* = 2.314, d*f* = 22, *P* = 0.0304, Fig. 2D).

After the traction test, we verified autism-like behaviours in VPA-exposed offspring. In the open field test, the numbers of centre grid squares (Welch-corrected, *t* = 3.266, d*f* = 14.74, *P* = 0.0053) crossed by VPA-exposed rats were reduced compared to those crossed by control rats, suggesting increased anxiety levels in VPA-exposed rats. Moreover, VPA-treated rats exhibited abnormal exploratory behaviours with fewer total grid squares crossed (Welch-corrected, *t* = 5,429, d*f* = 14.26, *P* < 0.0001) and less climbing (Welch-corrected, *t* = 5.375, d*f* = 13.55, *P* = 0.0001, Fig. 2E). In the self-grooming test, the VPA group took more time to groom than the control group (Welch-corrected, *t* = 2.863, d*f* = 11.41, *P* = 0.0149, Fig. 2F), revealing that VPA-treated rats exhibited more repetitive behaviours. The results of the juvenile social play test showed that VPA-treated rats exhibited a significant reduction in the time of social contact (*t* = 4.636, d*f* = 22, *P* = 0.0001), while the digging (Mann–Whitney *U* = 42.50, *P* = 0.0946) and attacking behaviours (Mann–Whitney *U* = 69, *P* = 0.9260, Fig. 2G) were not statistically significant. In the social stage of the three-chamber test (0–10 min), VPA-treated rats spent more time in the object chamber (B chamber) (*t* = 4.148, d*f* = 22, *P* = 0.0004) and interacting with the object (Welch’s correction, *t* = 3.700, d*f* = 13.21, *P* = 0.0026). Meanwhile, the VPA group appeared to lose interest in social interaction and took less time to explore in the stranger 1 chamber (A chamber) (*t* = 4.268, d*f* = 22, *P* = 0.0003) and to interact with stranger 1 (*t* = 2.888, d*f* = 22, *P* = 0.0085). In the social novelty trial (10–20 min), VPA-treated rats had an impaired preference for staying in the stranger 2 chamber (B chamber) (*t* = 6.084, d*f* = 22, *P* < 0.0001) and interacting with the stranger 2 (novel rat) (Mann–Whitney *U* = 4, *P* < 0.0001) and tended to stay in the stranger 1 chamber (*t* = 5.801, d*f* = 22, *P* < 0.0001, Fig. 2H). These behavioural tests showed that VPA-treated rats presented a motor coordination deficiency and autism-like behaviours and verified the feasibility of establishing a VPA-induced rat model of autism.

### The dynamic changes in neuronal apoptosis and synaptic dysplasia in VPA-exposed rats were in accordance with the temporal expression of PGRN

**Fig. 3:**
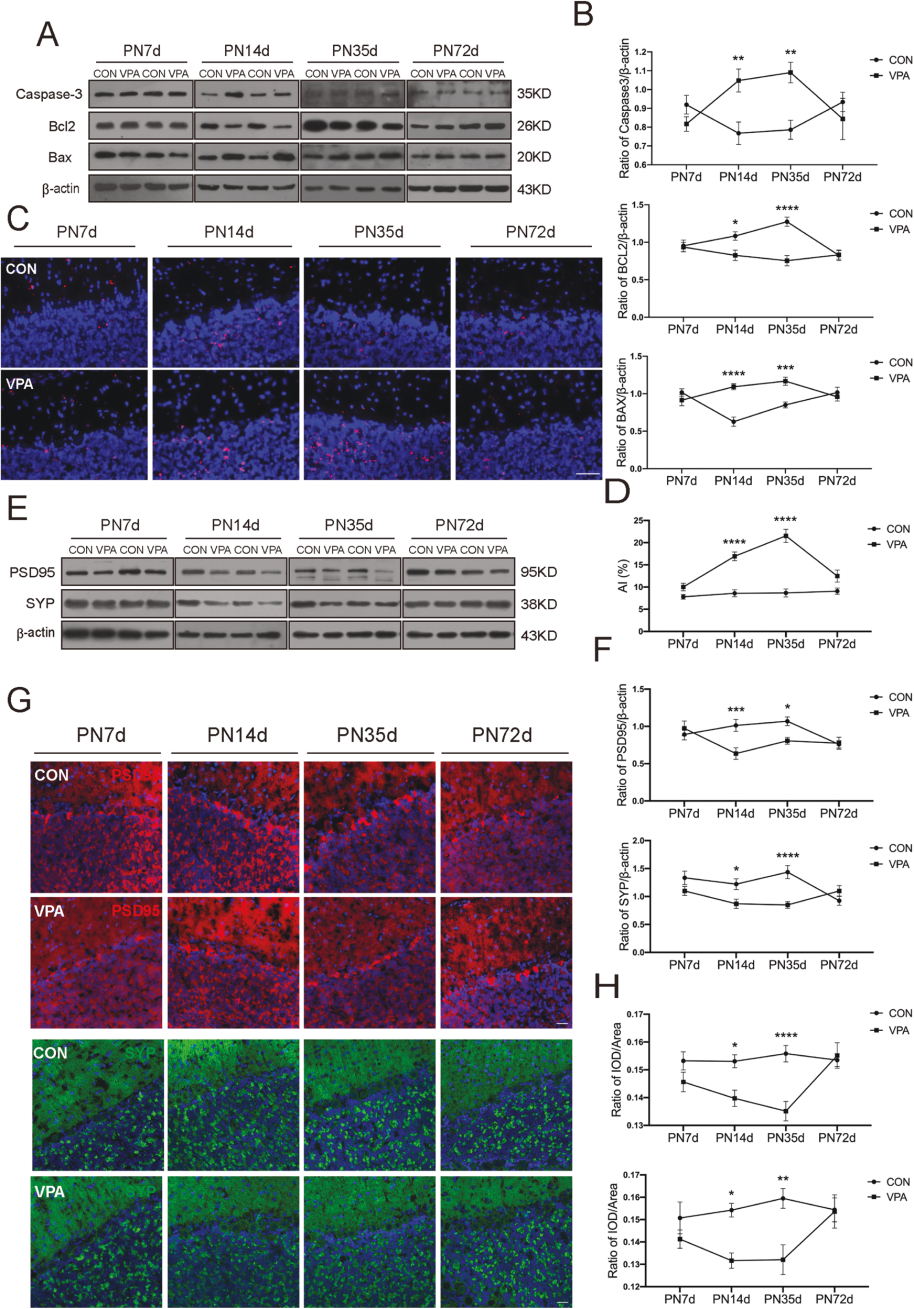
Dynamic changes of neuronal apoptosis and synaptic dysplasia in the cerebellum of VPA-induced rats. **A**, **B** Immunoblots and quantification exhibited expression levels of Caspase3, Bcl2, and Bax at different neurodevelopmental stages. **C**, **D** Representative images (bar = 50 μm) and quantification of TUNEL staining also showed increased neuronal apoptosis in the VPA group. **E**, **F** Western blotting showed synaptic markers, PSD95 and SYP, in the cerebellum. **G**, **H** Immunofluorescence staining (bar = 25 μm) exhibited consistent alterations in PSD95 and SYP protein expressions in VPA-induced rats. Data are expressed as mean ± SEM. Two-way ANOVA followed by Sidak post hoc. (*P < 0.05, **P < 0.01, ***P < 0.001, ****P < 0.0001, sample sizes (*n*): *n* = 5/group).

To study whether the temporal changes in PGRN protein expression mentioned above were related to disorders of neuronal apoptosis and synaptic formation/elimination, we detected neuronal apoptosis and the expression of synaptic markers at different time points with two-way ANOVA. We used Western blot analysis to detect the expression levels of apoptosis-related proteins, including Caspase-3, Bcl2 and Bax. According to the results, we found that the expression levels of Caspase-3 and Bax in the VPA group were higher than those in the control group at PND14 and PND35 (Caspase-3: *F*_(prenatal treat)1,120_ = 5.044, *P* = 0.0265, post hoc, *P* = 0.0089 for PND14, *P* = 0.0012 for PND35; Bax: *F*_(prenatal treat)1,118_ = 14.90, *P* = 0.0002, post hoc, *P* < 0.0001 for PND14, *P* = 0.0005 for PND35). Similarly, the expression of Bcl2 in VPA-treated rats was lower at PND14 and PND35 (*F*_(prenatal treatment)1,130_ = 17.36, *P* < 0.0001, post hoc, *P* = 0.0362 for PND14, *P* < 0.0001 for PND35, Fig. 3A, B). At the same time, the results of TUNEL staining showed that the number of apoptotic neurons in the cerebellum of VPA-treated rats was significantly greater than that in the cerebellum of control rats at PND14 and PND35 (*F*_(prenatal treat)1,112_ = 92.38, *P* < 0.0001, post hoc, *P* < 0.0001 for PND14, *P* < 0.0001 for PND35, Fig. 3C, D). Then, we detected the expression of the synaptic markers PSD95 and SYP. The results showed that the expression levels of PSD95 and SYP in the VPA-induced rats were markedly lower than those in the control group at PND14 and PND35 (Western blot: (PSD95: *F*_(prenatal treat)1,124_ = 7.131, *P* = 0.0086, post hoc, *P* = 0.001 for PND14, *P* = 0.0479 for PND35; SYP: *F*_(prenatal treat)1,118_ = 14.10, *P* = 0.0003, post hoc, *P* = 0.0437 for PND14, *P* < 0.0001 for PND35); immunofluorescence: (PSD95: *F*_(prenatal treat)1,112_ = 19.01, *P* < 0.0001, post hoc, *P* = 0.0173 for PND14, *P* < 0.0001 for PND35; SYP: *F*_(prenatal treat)1,108_ = 15.69, *P* = 0.0001, post hoc, *P* = 0.0125 for PND14, *P* = 0.0015 for PND35, Fig. 3E–H). From the above results, the temporal expression of PGRN was consistent with the occurrence of neuronal apoptosis and synaptic pathology in the cerebellum of VPA-treated rats. Thus, PGRN changes in VPA-treated rats may cause neuronal apoptosis and synaptic dysplasia.

### r-PGRN alleviated motor dysfunction and autism-related behaviours, and the PI3K inhibitor wortmannin reversed this effect in VPA-treated rats

Based on the above experimental results, we further verified the function of PGRN and explored the underlying signalling pathway, establishing four groups: the control group, VPA + Vehicle group, r-PGRN group, and r-PGRN + Wort group. All data were analysed with one-way ANOVA followed by a post hoc Tukey test or Kruskal–Wallis test (*n* = 11–13/group).

**Fig. 4:**
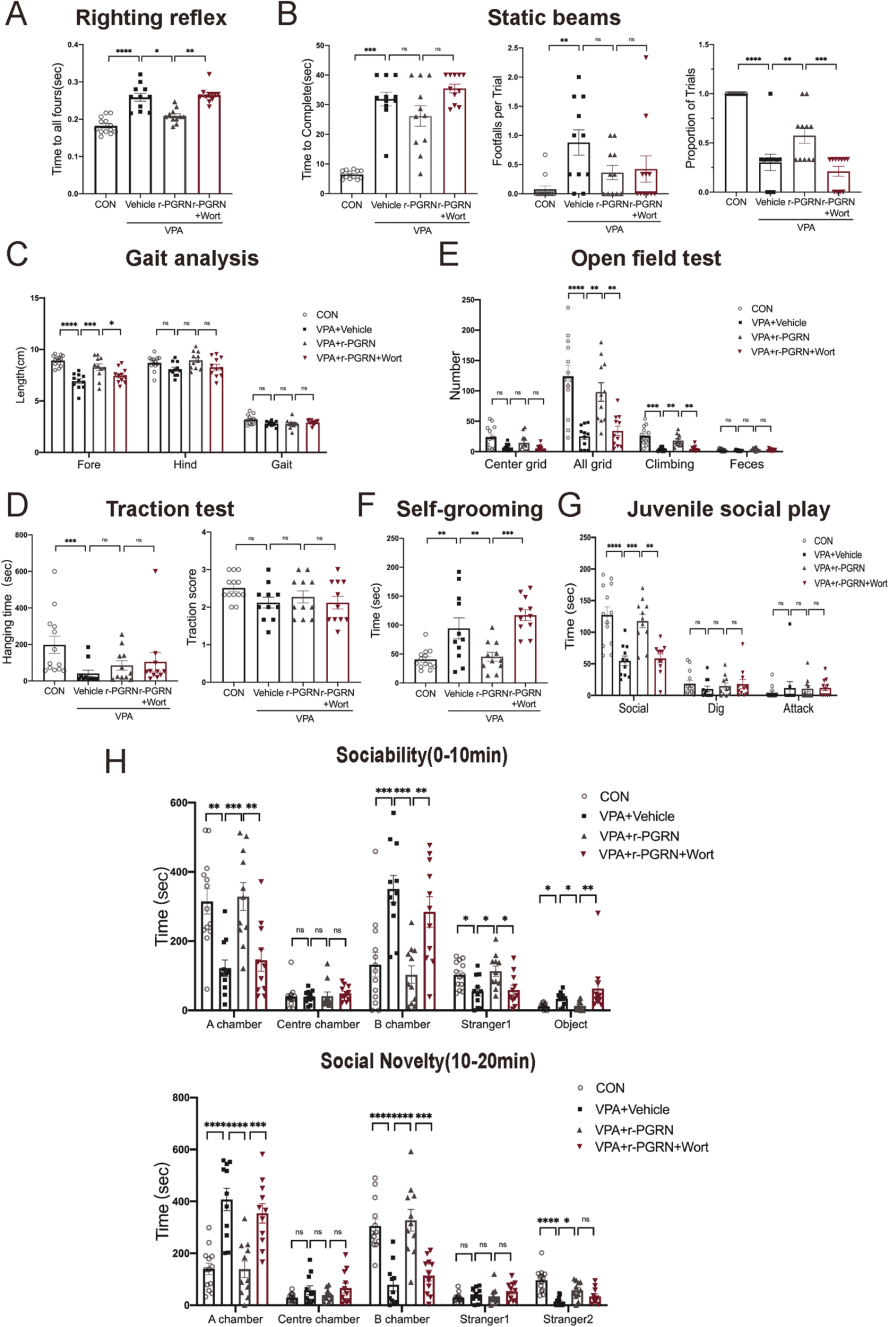
r-PGRN improved the defection of behaviours, and PI3K inhibitor wortmannin blocked this effect in VPA-induced rats. Cerebellum-related behaviours: righting reflex (**A**), static beams (**B**), gait analysis (**C**), traction test (**D**). Autism-like behaviours: open field test (**E**), self-grooming test (**F**), juvenile social play (**G**), and the three-chamber social test (**H**). Data are expressed as mean ± SEM. One-way ANOVA followed by Tukey’s post hoc (B_(Proportion of Trials)_, C_(Fore, Hind, Gait)_, D_(Traction score)_, E_(All grid, Faeces)_, F, G_(Social)_, H_(10 min: Stranger1, A chamber, B chamber)_, H_(20 min: A chamber, B chamber)_); post hoc Kruskal–Wallis test for multiple comparisons (A, B_(Time to Complete, Footfalls per Trial)_, D_(Hanging time)_, E_(Centre grid, Climbing)_, G_(Dig, Attack)_, H_(10 min: Object, Centre chamber)_, H_(20 min: Stranger1, Stranger2, Centre chamber)_). (ns no significant difference, *P < 0.05, **P < 0.01, ***P < 0.001, ****P < 0.0001, sample sizes (*n*): *n* (CON) = 13, *n* (VPA + Vehicle) = 11, *n* (r-PGRN) = 11, *n* (r-PGRN + Wort) = 11).

The results of the righting reflex showed that rats treated with r-PGRN took less time to turn over until all paws were flat than the VPA + Vehicle group, and the r-PGRN + Wort group spent more time than the r-PGRN group (Kruskal–Wallis statistic = 32.94, *P* < 0.0001; post hoc, *P* = 0.0278 for VPA + Vehicle vs. r-PGRN, *P* = 0.0081 for r-PGRN vs. r-PGRN + Wort, Fig. 4A). In the static beam test, the r-PGRN group had a significant increase in the success rate of passing the beam compared to the VPA group. The r-PGRN + Wort group exhibited a significant reduction in the success rate compared with the r-PGRN group (*F*_(3, 42)_ = 36.80, *P* < 0.0001; post hoc, *P* = 0.0061 for VPA + Vehicle vs. r-PGRN, *P* = 0.0004 for r-PGRN vs. r-PGRN + Wort, Fig. 4B). In the gait analysis test, the r-PGRN group had a significant increase in the forepaw stride lengths compared to the VPA group. The r-PGRN + Wort group showed shorter fore paw stride lengths in gait analysis tests than the r-PGRN group (*F*_(3, 42)_ = 15.22, *P* < 0.0001; post hoc, *P* = 0.0008 for VPA + Vehicle vs. r-PGRN, *P* = 0.0487 for r-PGRN vs. r-PGRN + Wort). The r-PGRN + Wort group showed a non-significant difference in all gait analysis tests compared to the r-PGRN group (Fig. 4C). The results of the traction test showed no significant difference between the r-PGRN group and VPA + Vehicle group or between the r-PGRN group and r-PGRN + Wort group (Hanging time: Kruskal–Wallis statistic = 14.91, *P* = 0.0019; post hoc, *P* = 0.7171 for VPA + Vehicle vs. r-PGRN, *P* > 0.9999 for r-PGRN vs. r-PGRN + Wort; Traction score: *F*_(3, 42)_ = 1.796, *P* = 0.1627, Fig. 4D).

Next, we observed the effects of exogenous recombinant PGRN on autism-related behaviours. In the open field test, the r-PGRN group exhibited more exploratory behaviours with a significant increase in the numbers of total grid squares crossed and the numbers of climbing bouts compared to the VPA + Vehicle group. The r-PGRN + Wort group crossed fewer grid squares (*F*_(3, 42)_ = 13.97, *P* < 0.0001; post hoc, *P* = 0.0021 for VPA + Vehicle vs. r-PGRN, *P* = 0.0084 for r-PGRN vs. r-PGRN + Wort) and had fewer climbing bouts than the r-PGRN group (Kruskal–Wallis statistic = 29.82, *P* < 0.0001; post hoc, *P* = 0.0055 for VPA + Vehicle vs. r-PGRN, *P* = 0.0061 for r-PGRN vs. r-PGRN + Wort, Fig. 4E). In the self-grooming test, PGRN-treated rats significantly reduced the time of grooming compared to the VPA group, while the r-PGRN + Wort group took more time to groom than the r-PGRN group (*F*_(3, 42)_ = 12.09, *P* < 0.0001; post hoc, *P* = 0.0093 for VPA + Vehicle vs. r-PGRN, *P* = 0.0002 for r-PGRN vs. r-PGRN + Wort, Fig. 4F). The juvenile social playtest results showed that the r-PGRN group spent significantly more time on social behaviours than the VPA + Vehicle group. In contrast, the r-PGRN + Wort group spent less time on social behaviours than the r-PGRN group (*F*_(3, 42)_ = 14.31, *P* < 0.0001; post hoc, *P* = 0.0006 for VPA + Vehicle vs. r-PGRN, *P* = 0.0013 for r-PGRN vs. r-PGRN + Wort, Fig. 4G). In the sociability trial of the three-chamber test, the r-PGRN group spent more time staying in the chamber containing the stranger1 rat and preferred to interact with stranger1 compared to the VPA + Vehicle group. Then, the r-PGRN + Wort group exhibited a defect in sociability compared to the r-PGRN group (B chamber: *F*_(3, 42)_ = 10.42, *P* < 0.0001; post hoc, *P* = 0.0002 for VPA vs. r-PGRN, *P* = 0.0074 for r-PGRN vs. r-PGRN + Wort; stranger1: *F*_(3, 42)_ = 5.267, *P* = 0.0036; post hoc, *P* = 0.0171 for VPA vs. r-PGRN, *P* = 0.0287 for r-PGRN vs. r-PGRN + Wort). In the social novelty trial, the r-PGRN group exhibited a significant preference to stay in the stranger 2 (novel rat) chamber and spent less time staying in the stranger 1 (familiar rat) chamber. The r-PGRN + Wort group spent more time staying in the familiar rat chamber (A chamber: *F*_(3, 42)_ = 17.56, *P* < 0.0001; post hoc, *P* < 0.0001 for VPA + Vehicle vs. r-PGRN, *P* = 0.0004 for r-PGRN vs. r-PGRN + Wort) and less time in the novel rat chamber (B chamber: *F*_(3, 42)_ = 18.59, *P* < 0.0001; post hoc, *P* < 0.0001 for VPA + Vehicle vs. r-PGRN, *P* < 0.0001 for r-PGRN vs. r-PGRN + Wort, Fig. 4H). These experimental results revealed that r-PGRN treatment in the cerebellum positively affected motor function deficits and autism-related behaviours, and wortmannin could reverse this effect in VPA-treated rats.

### r-PGRN reduced neuronal apoptosis and improved synaptic development, while the PI3K inhibitor wortmannin reversed this effect in VPA-treated rats

**Fig. 5:**
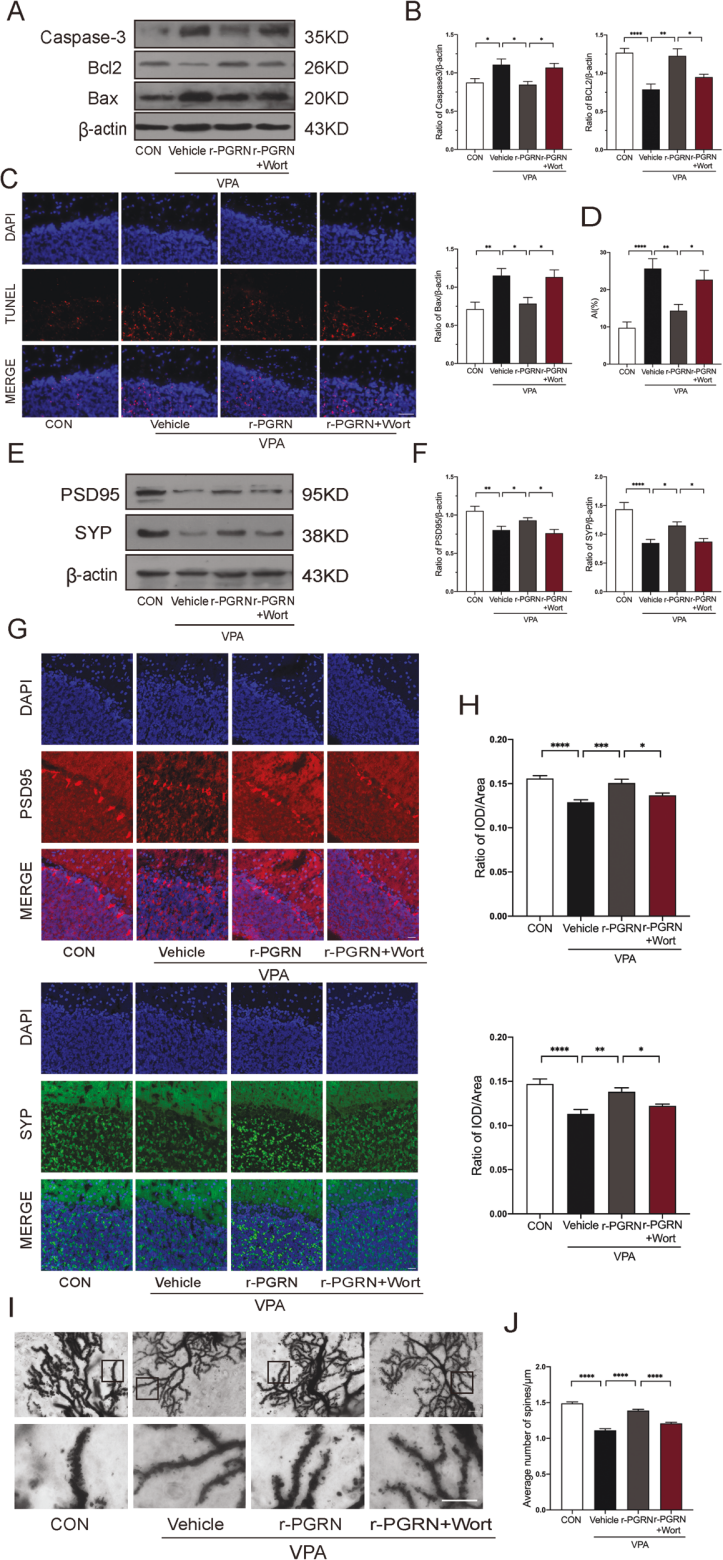
r-PGRN reduced neuronal apoptosis and improved synaptic development, while PI3K inhibitor wortmannin reversed this effect in VPA-induced rats. Representative blots (**A**) and quantification (**B**) of Caspase-3, BCL-2, and Bax in the four groups. **C**, **D** Representative images (bar = 50 μm) and quantification of TUNEL staining. Representative blots (**E**) and quantification (**F**) of synaptic markers such as PSD95 and SYP. **G**, **H** Immunofluorescence staining (bar = 25 μm) exhibited consistent changes in PSD95 and SYP protein expressions. Representative images of Purkinje cell dendrites (**I**) that were used for quantification of spines (bar = 10 μm) and quantification (**J**) of the number of spines. Data are expressed as mean ± SEM. One-way ANOVA followed by Tukey’s post hoc (B_(Caspase-3, Bax)_, D, F_(SYP)_, **H**); post hoc Kruskal–Wallis test for multiple comparisons (B_(Bcl2)_, F_(PSD95)_, **J**). (*P < 0.05, **P < 0.01, ***P < 0.001, ****P < 0.0001, sample sizes (*n*): *n* = 5/group).

Having demonstrated that PGRN could ameliorate abnormal behaviours in VPA-exposed rats, we investigated the neuroprotective effect of PGRN. In the four groups, we used Western blotting to assess the expression levels of apoptosis-related proteins such as Caspase-3, Bcl-2, and Bax. Moreover, TUNEL staining was used to calculate the number of apoptotic neuronal cells. One-way ANOVA revealed that the expression of Caspase-3 and Bax was significantly decreased and Bcl2 was higher in the r-PGRN group than in the VPA group. The results of TUNEL staining showed that the number of apoptotic neurons in the r-PGRN group was less than that in the VPA group. In contrast, the r-PGRN + Wort group exhibited the opposite trend compared to the r-PGRN group (Caspase-3: *F*_(3, 56)_ = 5.493, *P* = 0.0022; post hoc, *P* = 0.0102 for VPA + Vehicle vs. r-PGRN, *P* = 0.0373 for r-PGRN vs. r-PGRN + Wort; Bax: *F*_(3, 56)_ = 6.665, *P* = 0.0006; post hoc, *P* = 0.0253 for VPA + Vehicle vs. r-PGRN, *P* = 0.0370 for r-PGRN vs. r-PGRN + Wort; Bcl2: Kruskal–Wallis statistic = 27.54, *P* < 0.0001; post hoc, *P* = 0.0013 for VPA + Vehicle vs. r-PGRN, *P* = 0.0364 for r-PGRN vs. r-PGRN + Wort; TUNEL staining: *F*_(3, 56)_ = 11.75, *P* < 0.0001; post hoc, *P* = 0.0018 for VPA + Vehicle vs. r-PGRN, *P* = 0.0241 for r-PGRN vs. r-PGRN + Wort, Fig. 5A–D). The expression levels of PSD95 and SYP were significantly increased in the r-PGRN group but decreased in the r-PGRN + Wort group (Western blot: (PSD95: Kruskal–Wallis statistic = 21.49, *P* < 0.0001; post hoc, *P* = 0.0305 for VPA + Vehicle vs. r-PGRN, *P* = 0.0336 for r-PGRN vs. r-PGRN + Wort; SYP: *F*_(3, 60)_ = 12.43, *P* < 0.0001; post hoc, *P* = 0.0319 for VPA + Vehicle vs. r-PGRN, *P* = 0.0377 for r-PGRN vs. r-PGRN + Wort, Fig. 5E, F); immunofluorescence: (PSD95: *F*_(3, 56)_ = 14.07, *P* < 0.0001; post hoc, *P* = 0.0001 for VPA + Vehicle vs. r-PGRN, *P* = 0.0222 for r-PGRN vs. r-PGRN + Wort; SYP: *F*_(3, 56)_ = 11.57, *P* < 0.0001; post hoc, *P* = 0.0011 for VPA + Vehicle vs. r-PGRN, *P* = 0.0427 for r-PGRN vs. r-PGRN + Wort, Fig. 5G, H). Meanwhile, we observed the spine density in the four groups of the cerebellar Purkinje cells. The number of spines per μm was significantly decreased in the VPA group. The dendritic spine density of PGRN-treated rats was increased while it was reduced observably in the r-PGRN + Wort group (Kruskal–Wallis statistic = 191.7, *P* < 0.0001; post hoc, *P* < 0.0001 for CON vs. VPA + Vehicle, post hoc, *P* < 0.0001 for VPA + Vehicle vs. r-PGRN, *P* < 0.0001 for r-PGRN vs. r-PGRN + Wort). These results show that PGRN could cause a significant reduction in apoptosis and promote the development of synapses. The effects were reversed by wortmannin, indicating that PGRN mediates neuronal development via PI3K and subsequent downstream signalling.

### PGRN activated the PI3K/Akt/GSK-3β signalling pathway to regulate neuronal apoptosis and synaptic development in VPA-treated rats

**Fig. 6:**
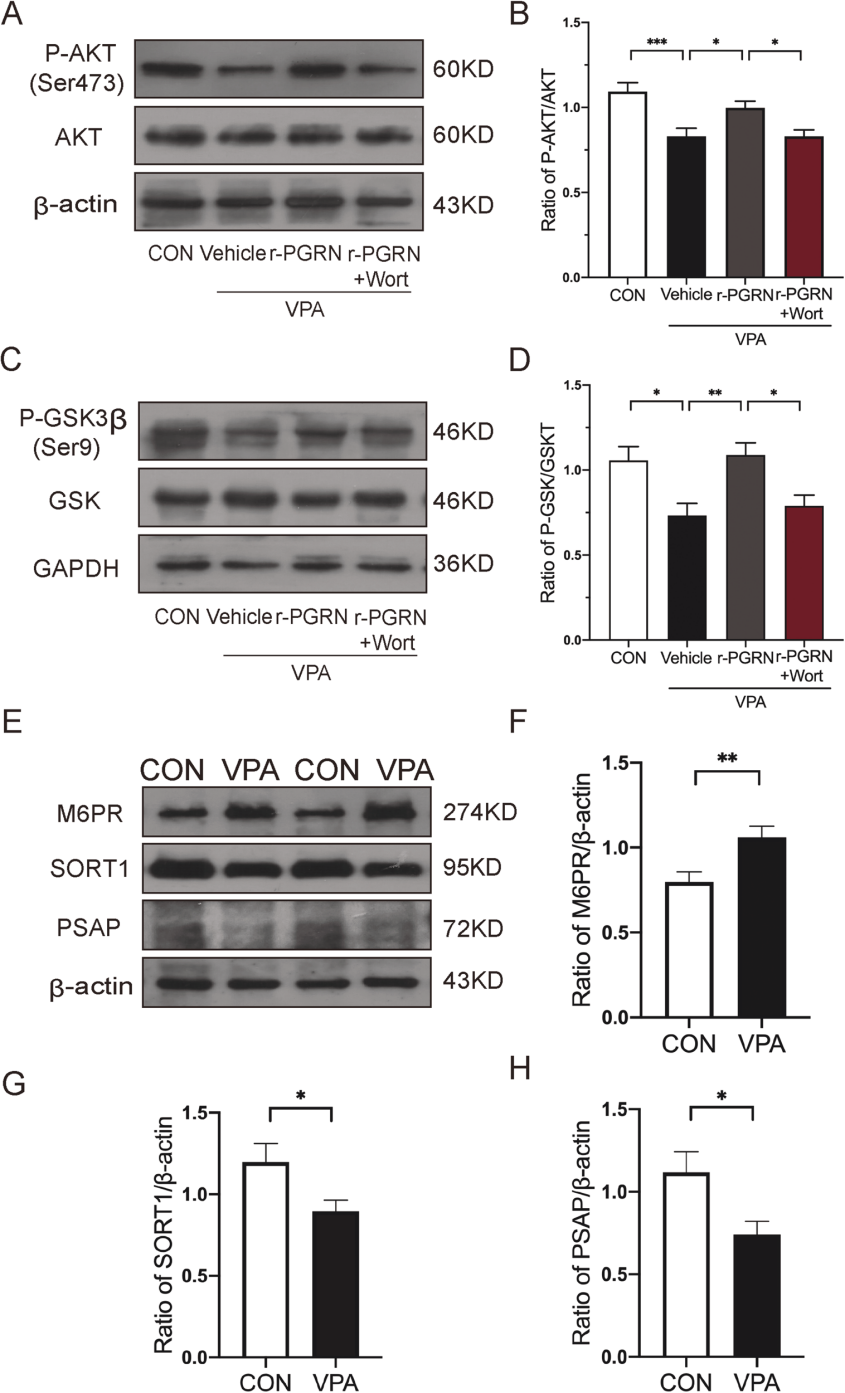
PGRN activated PI3K/Akt/GSK-3β signalling pathway and the expression of M6PR, SORT1 and PSAP in VPA-induced rats at PND35. Representative Western blots (**A**, **C**) and densitometric quantification (**B**, **D**) of p-Akt (Ser473), Akt, p-GSK-3β (Ser9) and GSK-3β expressions in the cerebellum. Representative Western blots (**E**) and densitometric quantification (**F**–**H**) of M6PR, SORT1 and PSAP. Data are expressed as mean ± SEM. One-way ANOVA followed by Tukey’s post hoc (**B**, **D**); Student’s *t* test (**F**, **H**); Mann–Whitney test (**G**). (*P < 0.05, **P < 0.01, ***P < 0.001, sample sizes (*n*): *n* = 5/group).

To prove our speculation, we used Western blotting to evaluate the expression of the downstream signalling proteins of PI3K. The PI3K/Akt/GSK-3β signalling pathway plays an essential role in neuronal survival and differentiation. The results showed that the expression levels of p-Akt (Ser473)/Akt and p-GSK-3β (Ser9)/GSK-3β in the VPA group were significantly lower than those in the control group. The expression levels of P-Akt/Akt and P-GSK-3β/GSK-3β were decreased in the r-PGRN + Wort group compared with the r-PGRN group, whereas the r-PGRN group showed meaningfully higher expression levels of P-Akt/Akt and P-GSK-3β/GSK-3β than the VPA group (P-Akt/Akt: *F*_(3, 56)_ = 8.625, *P* < 0.0001; post hoc, *P* = 0.0006 for CON vs. VPA + Vehicle, *P* = 0.0477 for VPA + Vehicle vs. r-PGRN, *P* = 0.0480 for r-PGRN vs. r-PGRN + Wort; P-GSK-3β/GSK-3β: *F*_(3, 60)_ = 6.266, *P* = 0.0009; post hoc, *P* = 0.0131 for CON vs. VPA + Vehicle, *P* = 0.0053 for VPA + Vehicle vs. r-PGRN, *P* = 0.0252 for r-PGRN vs. r-PGRN + Wort, Fig. 6A–D). Taken together, the evidence revealed that PGRN exerted a neuroprotective effect by modulating PI3K/Akt/GSK-3β signalling in VPA-exposed rats.

### PGRN level was regulated by PASP pathway independent of sortilin in VPA-treated rats

Recent studies suggest that PGRN is delivered into the lysosome for degradation mainly through two pathways, including the sortilin lysosomal pathway and the sortilin-independent lysosomal pathway. Based on this finding, we examined the expression levels of SORT1, PSAP and M6PR in VPA-treated rats with Western blot analysis. The results indicated that the expression of SORT1 decreased significantly compared with that in the control group (Mann–Whitney *U* = 62, *P* = 0.0357), illustrating that the endocytosis of PGRN was not mediated by sortilin. The results showed that the expression of PSAP decreased significantly compared with that in the control group (*t* = 2.524, d*f* = 28, *P* = 0.0176). Moreover, the expression level of M6PR in VPA-treated rats was higher than that in the control group (*t* = 2.907, d*f* = 30, *P* = 0.0068, Fig. 6E–H). Therefore, our data indicate that the PSAP pathway, the sortilin-independent lysosomal pathway, may be involved in regulating PGRN expression.

## Discussion

Because of the close relationship between PGRN and neural development, we investigated the role of PGRN in the VPA-induced rat model of autism. In this study, we proved that the expression of PGRN was significantly reduced in the cerebellum at postnatal day 14 (PND14) and in the prepubertal stage (PND35). Moreover, our results demonstrated for the first time that stereotactic injection with r-PGRN in the cerebellum improved behavioural deficits and neural development in VPA-treated rats. In addition, we explored the neuroprotective mechanism of r-PGRN mediated by the PI3K/Akt/GSK-3β signalling pathway, which is related to neuronal apoptosis and synaptic formation/elimination. Finally, we hypothesised that the downregulation of PGRN might be regulated by the PSAP pathway.

PGRN functions as an autocrine neuronal growth factor that has attracted significant attention in the neuroscience community following the recent discovery of PGRN mutations in some cases of FTLD. Our group previously demonstrated that abnormal spatiotemporal expression of PGRN is related to neurodevelopment impairment in VPA-induced autism models [23]. The expression of PGRN was elevated in the prefrontal cortex and somatosensory cortex at PND35 but was downregulated in the hippocampus at PND35 in VPA-exposed rats. Moreover, a recent study showed that PGRN-deficient mice displayed autism-like behaviours, including excessive self-grooming, which indicates repetitive stereotyped behaviour [17, 24]. Although only repetitive stereotyped behaviour is included in the diagnostic criteria for ASD, motor impairment is a fundamental feature in ASD [25]. It has been suggested that dyspraxia is a core ASD feature rather than a comorbid or associated disorder [26]. Our research detected for the first time that PGRN expression was significantly reduced in the cerebellum of VPA-induced autism rats. The expression level of PGRN in the cerebellum in other ASD models (such as genetic animal models) and ASD patients has not been reported. We also found that VPA-treated rats showed autistic core symptoms, such as reduced social preference, increased repetitive behaviours, and abnormal anxiety. In addition, animals exposed to VPA performed observably worse in each of the cerebellar tests in our study, consistent with a previous study [27].

Then, we verified that increasing the expression of PGRN in the cerebellum could ameliorate the behavioural abnormalities in VPA rats, especially motor function deficits. Our results clearly showed that r-PGRN injection could improve balance, gait, and motor coordination. However, it could not significantly reverse the hanging time and scores in the traction test. The traction test is a measure of muscle strength. The cerebellum plays an important role in regulating muscle strength [28]. However, grip strength is also related to whole-brain atrophy, white matter hyperintensities (WMHs) [29], and interactions between areas of the cortical grasping network [30]. VPA exposure possibly led to a reduction in muscle strength through other underlying mechanisms. Therefore, the injection of r-PGRN in the cerebellum did not reverse muscle strength significantly. The autism-related behaviour test showed that social interaction, repetitive behaviours, space exploration, social preference and social novelty were improved in r-PGRN-treated rats. However, in the open field test, there was no significant increase in the number of centre grid squares crossed in the r-PGRN group, indicating that PGRN injection could not effectively relieve the anxiety of rats. Anxiety symptoms are a common co-occurring condition in children with ASD [31]. Evidence supports multiple neurobiological hypotheses for the pathogenesis of anxiety, including abnormalities in neurotransmitter systems (serotonin, GABA, and norepinephrine), as well as the involvement of limbic circuits (particularly the amygdala) [32, 33]. Meanwhile, it has been reported that the imbalance between PGRN and TNFα contributes to thigmotaxis/anxiety caused by sleep deprivation [34]. Currently, the connections between the cerebellum and these factors are unknown. Therefore, further work exploring the exact mechanism of the unrecovered behaviour of anxiety in autism model rats is needed. Mostly, pharmacological agents for autism have limited therapeutic effects on repetitive and compulsive behaviours and may have adverse effects [35]. However, PGRN alleviated major autistic behaviours and motor function deficits, including repetitive, compulsive behaviours, but few severe adverse effects. Hence, PGRN has the potential to be a novel and valuable protein for human ASDs.

Although the pathogenesis of autism is not clear and definite, emerging evidence has proven that apoptotic mechanisms are involved in this disorder [36]. Apoptosis, also known as “programmed cell death,” is a vital mechanism that regulates the shape and size of the brain and regulates the appropriate wiring of developing neuronal networks [37]. However, pathological stimulation of apoptotic pathways may lead to neuroanatomic and neurodevelopmental abnormalities. Based on the theory of anatomic abnormalities in ASDs, it seems that there is excessive apoptosis in the childhood and adolescent autistic brain [38]. Several other studies also support that the expression of the anti-apoptotic protein Bcl-2 was significantly decreased, while caspase-3, a critical executioner of apoptosis, was increased in the cerebellum of autistic subjects [39, 40]. Accordingly, Western blot analyses revealed the same significant protein trend at PND14 and PND35, indicating increased apoptosis in the cerebellum. TUNEL staining also further confirmed that apoptosis was significantly increased in VPA-treated rats. PGRN, which is highly expressed in neurons, acts as a neurotrophic factor to enhance neuronal survival [41, 42]. Nevertheless, a direct neuroprotective impact of PGRN has not been investigated in autistic animal models. Our study found that r-PGRN treatment significantly decreased neuronal apoptosis in the cerebellum at PND35. Therefore, these results supported a neuroprotective role of PGRN in autistic rats. However, we cannot eliminate the possibility that other protective effects of PGRN, such as reducing inflammation, may also play a role in the neuroprotective effect [43, 44]. Thus, the link between different protective effects of PGRN also is considered in our other studies.

Synapses are information-processing units of the brain that structure the functional contact sites between neurons. Changes in the number and function of synapses can lead to variations in synaptic plasticity, thus contributing to cognitive and behavioural defects [45]. Both genetic and neurobiological evidence demonstrates that impairments in synapse formation and synaptic plasticity are widely recognised as a risk for in many brain disorders classified as synaptopathies, such as ASD, Alzheimer’s disease, and ADHD [46]. Studies have revealed that synapse formation and synaptic plasticity are decreased in autistic patients [47, 48]. Moreover, ASD, as a neurodevelopmental disorder, influences neural circuit establishment across diverse brain areas, among which the cerebellum has been identified [49]. Thus, the cerebellum appears to be a highly relevant encephalic region for studying the pathogenesis of ASD. We detected the expression of PSD95 and SYP, which are synaptic markers, in the cerebellum of VPA-exposed rats. We found that the expression of PSD95 and SYP was significantly decreased in VPA-exposed rats compared to controls. They all implicate synaptic strength, synaptic plasticity, and dendritic spine morphogenesis and serve as the synaptic number and density index during neurodevelopment [50]. Therefore, it is probable that abnormal expression of PSD95 and SYP may change synaptic plastic events that contribute to the synapse abnormalities associated with neurological disorders. PGRN has neurotrophic properties and plays an essential role in regulating neuronal connectivity. Activity-dependent secretion of PGRN can regulate synapse number and structure [51, 52]. To examine the function of PGRN in synaptic development, we tested the expression of PSD95 and SYP after VPA-treated rats were treated with r-PGRN. Our findings show that the expression levels of synaptic markers were significantly increased in the r-PGRN group, indicating that the synaptic number and density were rescued. Consequently, PGRN may rescue behavioural deficits by regulating synaptic development in the VPA-induced rat model of autism. The role of the cerebellum in ASD is an interesting topic. Previous studies have provided substantial evidence that the cerebellum is an important part of the brain circuits involved in disorders characterised by abnormal social behaviours [53]. Early cerebellar damage and/or defection in cerebellar development could impact a wide range of behaviours via the closed-loop circuits connecting the cerebellum with multiple cerebral cortical regions [54]. In addition, the cerebellum may be an upstream driver of the functionality and maturation of other cortical and subcortical brain structures involved in the core symptoms of ASD [49]. Future studies will be necessary to comprehend precisely how developmental synapse disruptions influence cerebrocerebellar circuits in ASD models.

Increasing investigations have demonstrated that PI3K/Akt signalling is an essential intracellular signalling system used by neurotrophic factors involved in neuron survival and differentiation [55] and implicated in the pathogenesis of autism [56]. GSK-3β, a kinase that is abundantly present in the brain, promotes neuronal apoptosis, and dysregulation of this kinase has a destructive effect on neurodevelopment [57]. Our study detected p-Akt (Ser473) and p-GSK-3β (Ser9) expression in the cerebellum of the VPA-induced rat model of autism. The expression levels of p-Akt and p-GSK-3β were found to be decreased in the VPA group. However, the above indicators were significantly increased in the r-PGRN group, in which neuronal apoptosis and synaptic development were improved. This result is consistent with recent reports that PGRN increased neuronal survival and neurite outgrowth [14] and activated the AKT/GSK-3β pathway [58]. We further investigated whether the neuroprotective effect of PGRN in the brain was completed through the PI3K/Akt/GSK-3β signalling pathway. After cerebellar injection of wortmannin to inhibit the PI3K/Akt pathway, the expression levels of p-AKT and p-GSK-3β in the PI3K inhibitor group were significantly decreased compared with those in the r-PGRN group, and all experimental indicators, including animal behaviours, were significantly different from those in the r-PGRN group. Therefore, our results identified that the addition of wortmannin reversed the activation of Akt and deactivation of GSK-3β by PGRN, further confirming that stimulation of PI3K/Akt/GSK-3β is involved in the neuroprotection of PGRN.

PGRN, the precursor of granulin peptides, is mainly delivered into lysosomes for degradation, and several membrane receptors are involved in this process [59]. However, the precise receptor that is responsible for PGRN-induced neurotrophic properties has not yet been identified. Sortilin has been identified as an essential protein that regulates the expression levels of progranulin by mediating the endocytosis of PGRN [60, 61]. However, research has demonstrated that PGRN does not necessitate sortilin binding for neuroprotective effects [62]. Zhou et al. (2015) were the first to report that PSAP carries PGRN along with it to lysosomes in both biosynthetic and endocytic pathways independent of sortilin, via the cation-independent mannose 6-phosphate receptor (M6PR) and low-density lipoprotein receptor-related protein 1 (LRP1) [63]. Furthermore, studies indicate that PSAP is a regulator of PGRN levels and oligomeric composition [64]. To further clarify the mechanism by which PGRN is expressed at low levels in the VPA-induced rat model of autism, we assessed the expression of sortilin, PSAP and M6PR. Our results further confirmed previous conclusions that the expression levels of sortilin and PSAP in VPA-exposed rats were significantly reduced, whereas M6PR was elevated compared to the control group. Therefore, sortilin is not the only neuronal receptor of PGRN. PSAP is an important factor related to PGRN, further supporting that the PGRN-PSAP interaction is likely relevant to the molecular mechanisms underlying autism. Nevertheless, additional studies, including studies of interventions that target PSAP expression, will be required to confirm whether and how PSAP plays a part in the regulation of PGRN.

Taken together, the findings from the present study demonstrated that PGRN could improve neural development via the activation of the PI3K/Akt/GSK-3β signalling pathway in a VPA-induced autism model. Meanwhile, we demonstrated that the expression level of PGRN might be regulated by the PSAP pathway independent of sortilin. In conclusion, the elucidation of the neuroprotective effects of PGRN in the present study adds to a growing literature suggesting the potentially positive role of this factor in neurological diseases. However, studies investigating the neuroprotective effects of PGRN in other autism models are required to extrapolate the therapeutic efficacy of PGRN.

## Supplementary information

WB supplementary
